# Arterial spectral waveform analysis in the prediction of diabetic foot ulcer healing

**DOI:** 10.1177/0267659120957849

**Published:** 2020-09-21

**Authors:** Pasha Normahani, Rishi Agrawal, Viknesh Sounderajah, Prodromos Tsinaslanidis, Patrick Musonda, Zaheer Mehar, Katarzyna Powezka, Vasiliki Bravis, Mohammed Aslam, Usman Jaffer

**Affiliations:** 1 Imperial Vascular Unit, Imperial College NHS Healthcare Trust, London, UK; 2 School of Medicine, Health Policy & Practice, University of East Anglia, London, UK; 3 West Middlesex University Hospital Diabetic Foot Service, Chelsea and Westminster Hospital NHS Foundation Trust, Isleworth, London, UK; 4 Department of Diabetes and Endocrinology, St Mary’s Hospital, Imperial College NHS Healthcare Trust, London, UK

**Keywords:** vascular ultrasound, arterial spectral waveform, peripheral arterial disease, diabetic foot

## Abstract

**Objective:**

We assessed the association between (1) severity of vessel wall calcification, (2) number of patent vessels at the ankle and (3) arterial spectral waveform features, as assessed on a focused ankle Duplex ultrasound (DUS), and healing at 12-months in a cohort of patients who had their diabetic foot ulcers conservatively managed.

**Research design and methods:**

Scans performed on 50 limbs in 48 patients were included for analysis. Patient health records were prospectively reviewed for 12-months to assess for the outcome of ulcer healing.

**Results:**

We identified that the number of waveform components, peak systolic velocity, systolic rise time and long forward flow as well as the number of vessels patent at the ankle on DUS, may be useful independent predictors of healing, as noted by the trend towards statistical significance.

**Conclusion:**

Arterial spectral waveform features may be useful in predicting the chance of diabetic foot ulcer healing.

## Introduction

Peripheral arterial disease (PAD) is an important independent predictor of lower limb ulcer healing in patients with diabetes. However, the natural course of patients with arterial disease and diabetic foot ulceration remains unclear. Although re-vascularisation has been shown to be an effective treatment strategy, limb salvage rates following conservative treatment are approximately 54%, whilst foregoing the risk profile inherent to interventional procedures.^1^ The ability to accurately predict the chance of wound healing would assist clinicians in the management of patients presenting with diabetic foot ulceration.

As such, wound classification systems such as WiFi have been validated for this purpose.^2^ Bedside tests assessing flow and perfusion to the foot have also been demonstrated to have some utility in stratifying risk.^3^ However, little is known regarding the usefulness of arterial spectral waveform analysis. Duplex Ultrasound (DUS) is well-established practice in determining the anatomical distribution of arterial disease and it has been postulated that the arterial spectral waveform at the ankle can provide useful information regarding the upstream and downstream state of the vasculature in addition to assessing the burden of arterial calcification.

In this study, we aim to evaluate whether there is an association between (1) severity of vessel wall calcification, (2) number of patent vessels at the ankle and (3) arterial spectral waveform features as assessed on a focused ankle DUS and healing at 12-months in a cohort of patients who have had their diabetic foot ulcers conservatively managed.

## Research design and methods

The study was approved by the National Research Ethics Committee (reference no. 17/LO/1447).

### Data collection

Data were collected as part of a previously published study exploring the effectiveness of a multi-centre training programme to teach focused DUS examination of the anterior and posterior tibial arteries at the level of the ankle for the detection of PAD in diabetes.^4^ As part of the training programme, consecutive patients presenting to diabetic foot clinics at a teaching hospital in London and a district general hospital in London were invited to take part in the study and provided informed consent. A fully qualified vascular scientist performed the scan on each patient. All scans performed in this study utilised the Mindray M7 (Shenzhen, China) Portable Ultrasound System with a linear 6 to 14 MHz transducer.

Data were collected regarding components of the waveform (monophasic, biphasic, triphasic, occluded), peak systolic velocities (PSV), the systolic rise time (RT), the presence or absence of spectral broadening, infilling of the spectral window and long forward flow in diastole. The latter three variables were only collected for mono- and bi-phasic waveforms. Additionally, data were collected regarding the number of patent vessels at the ankle (anterior and posterior tibial arteries) and the level of vessel wall calcification, which was classified as either mild/moderate or severe (mild = visible calcification, no acoustic shadowing; moderate = short segments of acoustic shadowing; severe = long segments of acoustic shadowing). Descriptions of duplex ultrasound parameters are presented in Table 1.

**Table 1.** Description of duplex parameters.

Feature	Description	Example	Description
Components	Monophasic	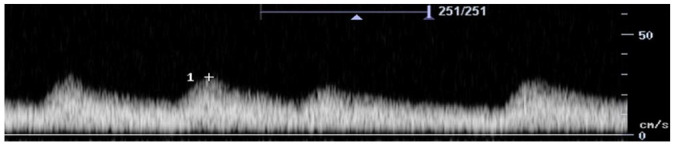	Monophasic waveform
	Biphasic	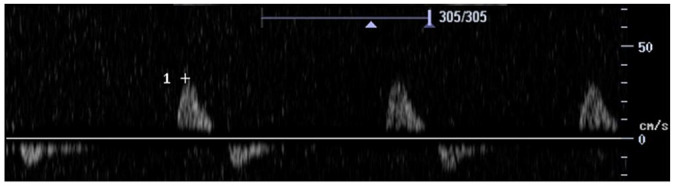	Biphasic waveform
	Triphasic	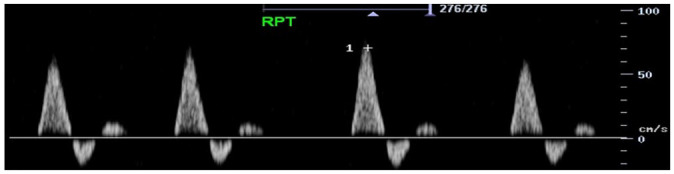	Triphasic waveform
Peak systolic velocity		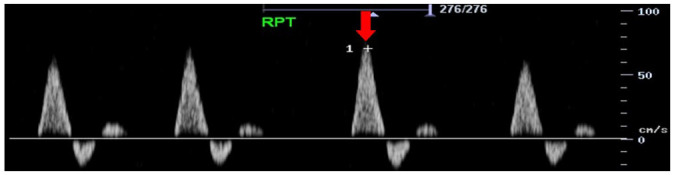	Measurement of peak systolic velocity
Systolic rise time		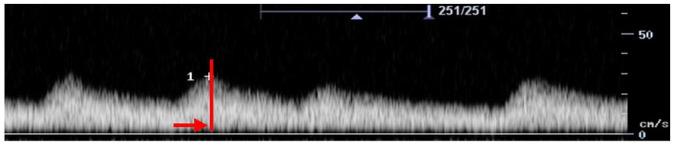	Measurement of systolic rise time
Spectral broadening	Yes	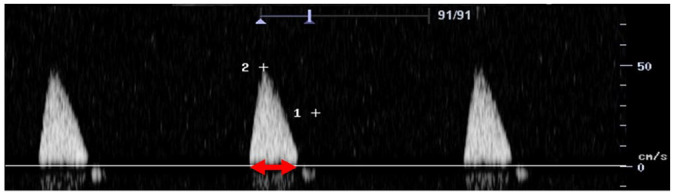	Broad biphasic waveform
	No	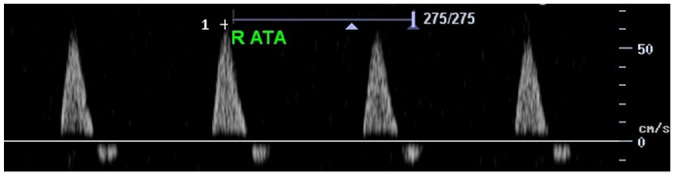	Narrow biphasic waveform
Infilling	Yes	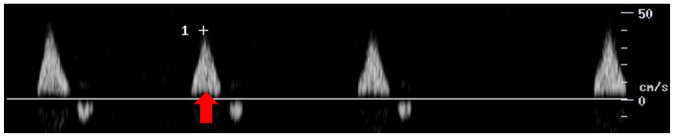	Loss of spectral window
	No	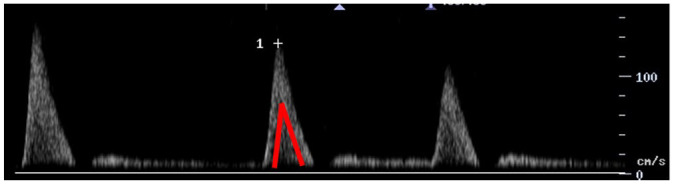	Spectral window is maintained
Long forward flow	Yes	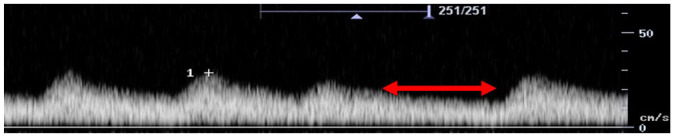	Monophasic waveform with long forward flow in diastole
	No	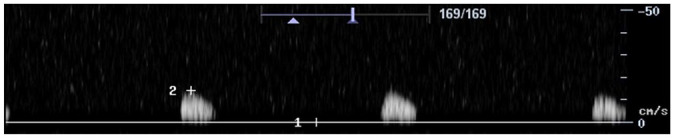	Monophasic waveform with absent forward flow in diastole
Vessel wall calcification	Mild	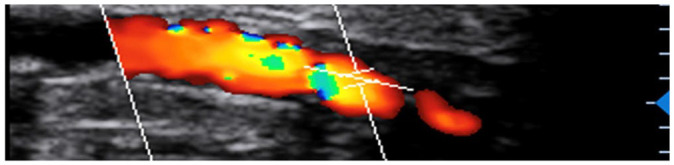	Visible calcification but no acoustic shadowing
	Severe	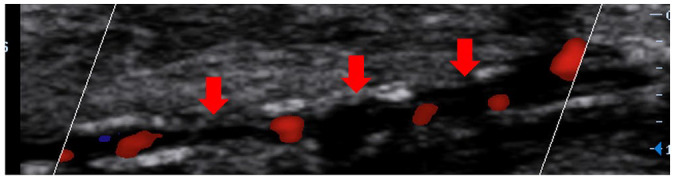	Long segments of acoustic shadowing

Patient electronic health records (eHR) were prospectively reviewed for 12-months following initial presentation to assess for the outcome of ulcer healing.

### Statistical analysis

For the purpose of analysis, the single best vessel (as assessed by the quality of the waveform: number of components followed by the absence of adverse features such as broadening, infilling and long forward flow) was used for analysis. The Shapiro-Wilk W-test and q-q plot were used to confirm that the continuous data were not normally distributed. Duplex ultrasound predictor variables were input into a binomial logistic regression model. Logit probabilities were converted to odds ratios. All analyses were performed using R (*R version 3.3.1; R Foundation for Statistical Computing, Vienna, Austria; https://www.r-project.org).*

## Results

A total of 83 limbs in 65 patients were scanned. Patients without active foot disease (n = 19) and those who had undergone re-vascularisation (n = 14) during the 12-month period following scanning were excluded from analysis. Therefore, results from scans performed on 50 limbs in 48 patients were included for analysis. Patient demographics are presented in Table 2. Twenty-seven out of 50 diabetic foot wounds (54%) healed within the 12-month follow-up period. Adjusted odds ratios for DUS predictor variables are presented in Table 3.

**Table 2.** Demographics of participants.

n	48
Gender = male (%)	32 (66.7)
Age (mean (SD))	67.6 (11.7)
Diabetes type = Type2 (%)	44 (91.7)
Duration of diabetes (mean (SD))	19.3 (10.1)
Renal failure = yes (%)	16 (33.3)
Retinopathy = yes (%)	26 (54.2)
Myocardial infarction = yes (%)	7 (14.6)
Angina = yes (%)	9 (18.8)
Heart failure = yes (%)	6 (12.5)
Stroke = yes (%)	6 (12.5)
Hypertension = yes (%)	34 (70.8)
Neuropathy = yes (%)	39 (81.2)

**Table 3.** Adjusted odds ratios for DUS predictor variables.

Factor	Odds ratio (95% CI)	p value
Waveform		
Tri/bi-phasic	Ref	
Monophasic	4.61e^+2^ (6.99e^−1^, 3.04e^+5^)	0.06
Peak systolic velocity (PSV)	1.07 (0.10, 1.15)	0.08
Systolic rise time (RT)	1.24e^−47^ (3.41e^−97^, 4.50e^+2^)	0.06
Calcium load		
Mild/moderate	Ref	
Severe	1.95e^−2^ (5.56e^−5^, 6.81)	0.19
Spectral broadening		
No	Ref	
Yes	4.29 (0.03, 548.79)	0.56
Long forward flow in diastole		
No	Ref	
Yes	38.18 (0.84, 1731.72)	0.06
Infilling of spectral window		
No	Ref	
Yes	1.92e^−13^ (∞,∞)	0.99
Number of vessels patent at the ankle		
2 vessels	Ref	
1 vessel only	7.47e^−3^ (3.06e^−5^, 1.82)	0.08

## Discussion

This is the first study to investigate the association between arterial spectral waveform features and the likelihood of diabetic foot ulcer healing. We included only conservatively managed patients because revascularisation after detecting PAD will ultimately influence the clinical outcome. This group allows us to exclusively investigate the role of ischaemia in wound healing without the influence of revascularisation. These patients received the same non-surgical care when compared to those patients undergoing revascularisation.

Although our study is underpowered, we have identified that certain spectral waveform features, such as the number of waveform components, PSV, RT and long forward flow in diastole as well as the number of vessels patent at the ankle on DUS, may be useful independent predictors of diabetic foot ulcer healing, as noted by the trend towards statistical significance. The ability to predict the chance of ulcer healing using arterial spectral waveform analysis is particularly attractive given that visual waveform analysis is one of the most accurate bedside diagnostic tool for the detection of PAD in people with diabetes.^5^ Therefore, risk assessment can be made at the time of presentation, which may in turn inform the urgency of onward vascular surgery referral, anatomical imaging and revascularisation, if required.

Better characterisation of flow profiles in the tibial vessels can give useful information regarding both the ‘up- and down-stream’ status of the arterial tree. Loss of pulsatility, long RT and low PSV are suggestive of severe upstream stenosis or occlusion and a long forward flow may be suggestive of distal vasodilatation in response to infection or ischaemia. In the future, we aim to further characterise waveform features that may be relevant to ulcer healing in order to develop new criteria that could be integrated into established or novel classification systems for more accurate grading of ischaemia. A previous study showed that integrating skin perfusion pressure assessment into the WIfI classification system resulted in more accurate staging.^6^

Although the presence of PAD is a major determinant of ulcer healing, other factors must also be considered when determining the chance of ulcer healing. The National Diabetic Foot Care Audit analysed data on over 27,000 ulcers and identified 15 important variables including the presence of PAD, size and number of ulcers, gender, inactive charcot, duration of diabetes, acute comorbidities and time to first expert assessment.^7^ Evidence is also emerging that diligent wound care, including regular debridement, dressings and offloading are also important predictors of healing.^8,9^
